# Geriatric Conditions and Prescription of Vitamin K Antagonists vs. Direct Oral Anticoagulants Among Older Patients With Atrial Fibrillation: SAGE-AF

**DOI:** 10.3389/fcvm.2019.00155

**Published:** 2019-10-30

**Authors:** David D. McManus, Catarina Kiefe, Darleen Lessard, Molly E. Waring, David Parish, Hamza H. Awad, Francesca Marino, Robert Helm, Felix Sogade, Robert Goldberg, Robert Hayward, Jerry Gurwitz, Weijia Wang, Tanya Mailhot, Bruce Barton, Jane Saczynski

**Affiliations:** ^1^Department of Population and Quantitative Health Sciences, University of Massachusetts Medical School, Worcester, MA, United States; ^2^Cardiology Division, Department of Medicine, University of Massachusetts Medical School, Worcester, MA, United States; ^3^Department of Allied Health Sciences, University of Connecticut, Mansfield, MA, United States; ^4^Department of Community Medicine/Internal Medicine, Mercer University School of Medicine, Macon, GA, United States; ^5^Department of Medicine, Boston University School of Medicine, Boston, MA, United States; ^6^Department of Medicine, Mercer University School of Medicine, Macon, GA, United States; ^7^Department of Electrophysiology, Kaiser Permanente Santa Clara Medical Center, Santa Clara, CA, United States; ^8^Division of Geriatric Medicine, University of Massachusetts Medical School, Worcester, MA, United States; ^9^Department of Pharmacy and Health System Sciences, Northeastern University, Boston, MA, United States; ^10^Montreal Heart Institute Research Center, Montreal, QC, Canada

**Keywords:** atrial fibrillation, anticoagulation, frailty, social isolation, older adults

## Abstract

**Background:** Geriatric conditions are common among patients with atrial fibrillation (AF) and relate to complications of oral anticoagulation (OAC).

**Objective:** To examine the prevalence of geriatric conditions among older patients with AF on OAC and relate type of OAC to geriatric conditions.

**Methods:** Participants had a diagnosis of AF, were aged ≥65 years, CHA_2_DS_2_VASC ≥ 2, and had no OAC contraindications. Participants completed a 6-component geriatric assessment that included validated measures of frailty (CHS Frailty Scale), cognitive function (MoCA), social support (MOS), depressive symptoms (PHQ9), vision, and hearing. Type of OAC prescribed was documented in medical records.

**Results:** 86% of participants were prescribed an OAC. These participants were on average aged 75.7 (*SD*: 7.1) years, 49% were women, two thirds were frail or pre-frail, and 44% received a DOAC. DOAC users were younger, had lower CHA_2_DS_2_VASC and HAS-BLED scores, and were less likely to be frail. In Massachusetts, pre-frailty was associated with a significantly lower odds of DOAC vs. VKA use (*OR* = 0.64, 95%CI 0.45, 0.91). Pre-frailty (*OR* = 0.33, 95%CI 0.18–0.59) and social isolation (*OR* = 0.38, 95%CI 0.14–0.99) were associated with lower odds of DOAC receipt in patients aged 75 years or older. Social isolation was associated with higher odds of DOAC use (*OR* = 2.13, 95%CI 1.05–4.29) in patients aged 65–74 years.

**Conclusions:** Geriatric conditions were common and related to type of OAC prescribed, differentially by age group. Research is needed to evaluate whether a geriatric examination can be used clinically to better inform OAC decision-making in older patients with AF.

## Introduction

Stroke prevention is central to atrial fibrillation (AF) treatment, and guidelines support use of oral anticoagulants (OAC) for AF patients at elevated risk for stroke (1). Three out of four AF patients aged 65 and older meet guideline criteria for OAC treatment (2).

Historically, use of an OAC for stroke prevention in AF meant use of a vitamin K antagonist (VKA). This treatment often requires frequent testing and dosing changes, since fluctuations in VKA response can result from diet, comorbid diseases, genetic variations, and drug-drug interactions. Four direct oral anticoagulants (DOACs) have been approved by the FDA for stroke prevention in non-valvular AF. These include: dabigatran, a direct thrombin inhibitor, and three factor Xa inhibitors, rivaroxaban (3), apixaban, and edoxaban (4). Recent AF management guidelines suggest that these agents be considered as first-line therapy for AF (5).

Despite the inclusion of older patients in several clinical trials comparing DOACs vs. VKA (6–8), many older patients were excluded due to comorbid illnesses. Since advanced age and common comorbidities may alter the pharmacokinetics of DOACs, the decision to prescribe a VKA or DOAC in “real-world” patients remains a conundrum. Although post-market surveillance studies show similar outcomes among DOAC and VKA-treated patients, few studies have examined differences in factors related to aging that strongly impact treatment outcomes (9). Conditions common in older patients, such as cognitive impairment, frailty, and social isolation adversely impact patient outcomes and are increasingly recognized as important components of prescribing decision making (10–17). In response to accumulating data that psychosocial factors influence treatment outcomes in older patients, AHA/ACC Guidelines for AF Management (1), as well as the AHA/ACC guidelines for other cardiovascular conditions, such as AMI, include consideration of CI, depression and social support in patient management (18). Also, geriatrics-specific guidelines for acute coronary care were developed by the AHA and ACC in 2007 (19), further highlighting the increasing attention to age-related factors in the management and outcomes of CVD. Although several of these conditions have been examined for their association with AC prescribing (20), whether type of AC prescribed varies according to psychosocial and geriatric conditions has not been examined.

Using data from the ongoing Systematic Assessment of Geriatric Elements in Atrial Fibrillation (SAGE-AF) prospective cohort study, we examined the characteristics of patients treated with DOACs vs. VKAs in a “real-world” cohort of older patients with AF. The objective of this study was to examine the prevalence of geriatric conditions among older patients with AF on OAC and relate type of OAC to geriatric conditions. We hypothesized that the presence of geriatric conditions would be associated with lower odds of receiving DOACs vs. VKAs. Further, we hypothesized that geriatric conditions would have different associations with prescribing patterns in younger vs. older patients with AF.

## Materials and Methods

SAGE-AF is an ongoing study of AF, OAC treatment, and relations between geriatric conditions and clinical outcomes in adults aged 65 years and older. Consenting participants completed a comprehensive baseline geriatric assessment, a structured interview, and review of their medical records.

To be eligible for SAGE-AF, participants must have: (1) been scheduled for an ambulatory care visit at a practice in Massachusetts or Georgia, (2) had a history of AF documentation on an electrocardiogram, Holter monitor, clinic note, or hospital record, and (3) had a CHA_2_DS_2_VASC risk score ≥2 (indication for OAC) (2). Participants were not eligible for enrollment if they had documentation of an OAC contraindication, had an indication for OAC other than AF, did not demonstrate capacity to provide informed consent (21), did not speak English, had a planned invasive procedure with possible uncontrollable bleeding, were pregnant, were in prison, or were unwilling or unable to participate in follow-up visits at their study sites.

All participants received an invitation to participate before their clinic visit. All participants provided written consent, and all study protocols were approved by the respective Institutional Review Boards. Between June 2016 and August 2018, 1,244 patients were enrolled.

All SAGE-AF participants had a medical history and physical examination performed in the context of their routine care. Trained study staff abstracted all demographic, clinical, treatment, and laboratory characteristics from participants' medical records. All participants underwent a comprehensive interview that included a 6-component geriatric assessment using validated measures of frailty, cognitive function, social support, depressive symptoms, vision, and hearing. Frailty was assessed using the Cardiovascular Health Survey (CHS) frailty scale (22), a biological model of frailty based on five components: weight loss/shrinking; exhaustion; low physical activity (Minnesota Leisure Time Activity questionnaire) (23); slow gait speed (15-foot timed walk); and weakness (grip strength). Each element receives a single point and the frailty index ranges from 0 to 5. Based on the CHS scoring guidelines (22), a participant is frail if 3 or more criteria are present, pre-frail if 1 or 2 criteria are present, and not frail if 0 criteria are present.

To assess cognition, participants completed the Montreal Cognitive Assessment Battery (MoCA), a 30-item screening tool designed to assist care healthcare providers in detecting mild cognitive impairment. A cut-point of 23 was used to classify cognitive impairment (24). The 6-item Social Network Scale was used to assess the participants' social networks and a cut-point of <12 was used to define social isolation (25). This measure of social isolation reflects social connectedness; whether a person has friends or family members to talk to about important issues or to call when they need help. Social isolation is linked to higher rates of disability, poorer recovery from illness and death (26–28). The 9-item Patient Health Questionnaire (PHQ-9) was used to assess depressive symptoms with a score of five used as a cut-point for depressive symptoms (29). Patients self-report vision and hearing impairments (30, 31).

## Statistical Analysis

We compared the characteristics of SAGE-AF participants who received OAC according to type of OAC (DOAC vs. VKA) using analysis of variance for continuous variables and *chi square* tests for categorical variables. We examined which geriatric elements (independent variables: cognitive function, frailty, social isolation, vision impairment, hearing impairment, depression) were associated with OAC treatment type using adjusted logistic regression analysis. In these analyses, we adjusted for each geriatric element and then additionally for clinical and demographic factors identified from univariate analyses as being associated with treatment type at the *p* < 0.15 level. Differences in the clinical and treatment characteristics were noted between participants from Massachusetts and Georgia. As such, we conducted stratified regression analyses by study site and adjusted for factors associated with OAC type. We also stratified our regression analyses by age (65–74 years vs. ≥75 years) since we were interested in whether geriatric conditions influenced OAC prescription choice differently among older and younger participants.

## Results

86% of the 1244 SAGE-AF participants were prescribed an OAC. Of the participants prescribed an OAC, we excluded 15 participants missing data on one or more of the variables included in the analysis, resulting in an analytic sample of 1,064 older patients with AF prescribed an OAC.

Of these participants, 44% were prescribed a DOAC and 56% were prescribed VKA. Among those prescribed a DOAC, the majority received apixaban (*n* = 238) and rivaroxaban (*n* = 184), with a minority of participants receiving dabigatran (*n* = 40) or edoxaban (*n* = 4). There was a high burden of cardiovascular and non-cardiovascular comorbidities, including prior bleeding and geriatric conditions (Table 1). Women comprised about half of the study sample, 85% were white, two-thirds were frail or pre-frail, and 22% had suffered a fall in the last 6 months. Differences in the overall proportion of DOAC treated participants and factors associated with type of OAC prescribed were noted in Massachusetts vs. Georgia (Supplemental Table 1).

**Table 1:** Characteristics of older adults with atrial fibrillation on oral anticoagulation, according to type of oral anticoagulation: SAGE-AF, 2016–2018.

**Characteristic**	**Direct oral anticoagulant** **(*n* = 466)**	**VKA** **(*n* = 598)**	***p*-value**
**DEMOGRAPHIC CHARACTERISTICS**			
**Age**			
65–74 years	251 (53.9)	268 (44.8)	<0.001
75–84 years	178 (38.2)	223 (37.3)	
85 years and older	37 (7.9)	107 (17.9)	
Female	228 (48.9)	297 (49.7)	0.81
**Race/Ethnicity**			
White	380 (81.6)	522 (87.3)	<0.01
Non-White	86 (18.5)	76 (12.7)	
**Marital status^a^**			
Married or living as married	266 (57.1)	327 (54.7)	0.73
Not Married	193 (41.4)	261 (43.7)	
**Education^b^**			
High school/GED or less	45 (9.7)	45 (7.5)	0.22
Some college	209 (44.9)	310 (51.8)	
College graduate	75 (16.1)	83 (13.9)	
Graduate degree	130 (27.9)	150 (25.1)	
**Household income^c^**			
>= 50,000	208 (52.0)	224 (46.0)	0.08
**Insurance status^d^**			
Commercial/HMO/PPO	56 (12.0)	128 (21.4)	<0.01
Medicare	350 (75.1)	422 (70.6)	
Other	59 (12.7)	46 (7.7)	
**CLINICAL CHARACTERISTICS**			
**AF type**			
Paroxysmal	298 (64.0)	302 (50.5)	<0.001
Persistent	118 (25.3)	179 (29.9)	
Permanent	19 (4.1)	50 (8.4)	
Unknown	31 (6.7)	67 (11.2)	
CHA_2_DS_2_VASC score (M, *SD*)	4.3 (1.6)	4.7 (1.6)	<0.01
HAS-BLED score (M, *SD*)	2.8 (1.0)	3.0 (1.0)	<0.05
AFEQT score (M, *SD*)	78.2 (18.9)	80.5 (16.9)	<0.05
Bothered^e^ by ≥1 AF symptom in the past 4 weeks	61 (13.2)	55 (9.2)	<0.05
**Medical history**			
Acute myocardial infarction	89 (19.1)	123 (20.6)	0.55
Alcohol abuse/dependency	165 (35.4)	168 (28.1)	<0.05
Anemia	122 (26.2)	212 (35.5)	<0.01
Bleeding	97 (20.8)	112 (18.7)	0.4
Chronic kidney disease	115 (24.7)	195 (32.6)	<0.01
Chronic lung disease	123 (26.4)	145 (24.3)	0.42
Diabetes	140 (30.0)	166 (27.8)	0.41
Heart failure	159 (34.1)	250 (41.8)	<0.05
Hyperlipidemia	354 (76.0)	495 (82.8)	<0.01
Hypertension	415 (89.1)	551 (92.1)	0.08
Implantable cardiac device	163 (35.0)	203 (34.0)	0.73
Peripheral vascular disease	57 (12.2)	93 (15.6)	0.12
Stroke	40 (8.6)	68 (11.4)	0.13
Creatinine (mg/dL) M (*SD*)	1.05 (0.37)	1.15 (0.65)	<0.05
Hemoglobin (g/dL) M (*SD*)	13.1 (1.9)	13.1 (1.8)	0.96
**TREATMENT CHARACTERISTICS**			
Aspirin	104 (22.3)	204 (34.1)	<0.001
Clopidogrel	30 (6.4)	25 (4.2)	0.07
**OVERALL TREATMENT SATISFACTION**			
Extremely satisfied	203 (43.8)	286 (48.0)	0.41
Very satisfied	147 (31.7)	187 (31.4)	
Somewhat satisfied	61 (13.2)	64 (10.7)	
Satisfied or less than satisfied	53 (11.4)	59 (9.9)	
**PSYCHOSOCIAL CHARACTERISTICS**			
Fall in past 6 months	91 (19.5)	143 (23.9)	0.09
Anxiety^f^	115 (24.7)	137 (22.9)	0.50
Living Alone	128 (27.5)	167 (27.9)	0.87
Independence (IADLs)	6.8 (0.9)	6.6 (1.1)	<0.05
Confident in physician interactions^g^	302 (66.8)	374 (64.3)	0.39
**Practice type**			
Cardiologist	139 (29.8)	349 (58.4)	<0.001
EP	319 (68.5)	233 (39.0)	
Internist	8 (1.7)	16 (2.7)	

a *n = 17 missing Marital Status*.

b *n = 17 missing Education*.

c *n = 177 missing household income*.

d *n = 3 missing Insurance Status*.

e *quite/extremely/very bothered with symptoms*.

f *anxiety GAD7 <= 5*.

g *defined based on a PEPPI score of >45*.

**Figure 1:**
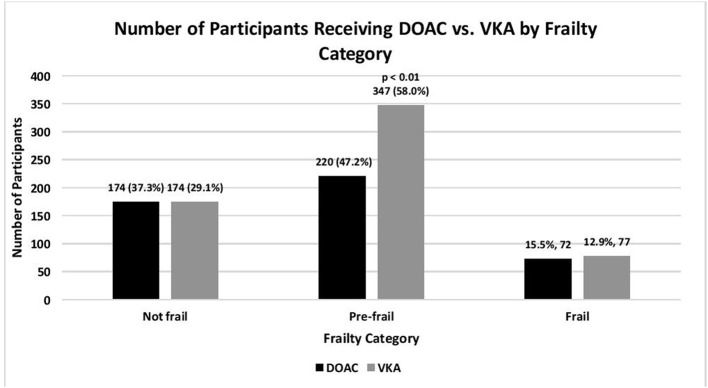
Number of older adults with atrial fibrillation on oral anticoagulation, according to type of oral anticoagulation, and frailty category: SAGE-AF, 2016-2018. Not frail, 0 impairments; Pre-frail, 1–2 impairments; Frail, 3–5 impairments (impairments include unintentional weight loss, weakness, exhaustion, slow gait, low physical activity) as defined by the CHS Frailty Scale (22).

Geriatric conditions, including frailty, depression, cognitive impairment, social isolation, and visual and hearing impairments were common (Table 1, Figure 1). Given the high rate of cardiovascular comorbidity, study participants were at high risk for thromboembolic, and bleeding complications based on their CHA_2_DS_2_VASC and HAS-BLED risk scores. Slightly more than three quarters (78%) of study participants reported being satisfied or very satisfied with their overall treatment.

In comparison to participants receiving VKA, participants treated with DOACs were, on average, younger, and had lower average stroke and bleeding risk scores (Table 1). Notably, participants treated with DOACs were more likely to have paroxysmal AF, have had a symptomatic episode in the last 4 weeks, have seen a cardiologist or cardiac electrophysiologist, and have reported lower OAC burden and higher disease-specific quality of life (32). Finally, participants receiving DOACs were less likely to be frail or pre-frail and had greater independence in activities of daily living but were more likely to report social isolation.

Among participants enrolled in Massachusetts, frail, pre-frail, and cognitively impaired status were associated with 40, 46, and 35% lower odds, respectively, of being treated with DOAC (Table 2). After adjustment for stroke and bleeding risk scores as well as other factors associated with type of OAC, pre-frail status was associated with a significantly reduced odds of being prescribed a DOAC (*OR* = 0.64, 95% CI 0.45, 0.91; Table 2). When we stratified our analyses by age, pre-frail status, and social isolation were associated with a more than 60% lower odds of DOAC-receipt in older (>75 years) participants enrolled in Massachusetts (Table 3). In contrast, low social isolation was associated with an approximately 2-fold higher rate of DOAC receipt among younger (65–74 years) participants enrolled in Massachusetts (Table 3). The sample size in Georgia was not large enough to examine these associations among patients in Georgia.

**Table 2:** Receipt of DOAC vs. VKA by geriatric element status among older adults with atrial fibrillation treated with oral anticoagulation enrolled in Massachusetts (*n* = 818): SAGE-AF, 2016–2018.

			**Odds of receiving a DOAC**			
**Geriatric elements**	**Received** **DOAC, *N* (%)**	***p*-value**	**M1 unadjusted OR** **(95% CI)**	***p*-value**	**M2 adjusted OR** **(95% CI)**	***p*-value**
Frailty						
Frail	29 (10.4)	<0.01	0.60 (0.37, 0.99)	0.41	0.88 (0.48, 1.61)	0.74 <0.05
Pre-frail	126 (45.3)		0.54 (0.39, 0.74)	<0.05	0.64 (0.45, 0.91)	
Not frail	123 (44.2)		(Reference)		(Reference)	
Cognitive impairment	89 (32.0)	<0.05	0.65 (0.48, 0.89)	<0.01	0.95 (0.67, 1.34)	0.76
Social isolation	28 (10.1)	0.97	1.01 (0.62, 1.63)	0.97	1.11 (0.66, 1.86)	0.70
Visual impairment	82 (29.5)	0.46	0.89 (0.65, 1.22)	0.46	1.02 (0.72, 1.44)	0.91
Hearing impairment	103 (37.1)	0.57	0.92 (0.68, 1.24)	0.57	1.03 (0.75, 1.43)	0.85
Depression	65 (23.4)	0.17	0.79 (0.57, 1.11)	0.17	0.79 (0.53, 1.19)	0.27

*M1, Unadjusted logistic regression model for each geriatric element (main independent variables): frailty, cognitive impairment, social isolation, sensory impairments (visual and hearing), depression; M2, M1 additionally adjusted for risk scores (CHA_2_DS_2_VASC, HAS-BLED), education, insurance, AF type, AFEQT score, hyperlipidemia, alcohol abuse, anemia, CKD, aspirin use, fall in past 6 months, IADL, provider type, frailty, and cognitive impairment. Age was accounted for via CHA_2_DS_2_VASC*.

**Table 3:** Receipt of DOAC vs. VKA by geriatric element status among older adults with atrial fibrillation treated with oral anticoagulant enrolled in Massachusetts, further stratified by age (75-years cutoff): SAGE-AF, 2016–2018.

**Geriatric elements**	**DOAC *N* (%)**	***p*-value**	**M1 unadjusted OR (95% CI)**	***p*-value**	**M2 adjusted OR (95% CI)**	***p*-value**
**<75 (*****N*** **= 406)**						
Frailty						
Frail	12 (7.4)		0.60 (0.28, 1.26)	0.27	0.84 (0.35, 2.01)	0.73
Pre-Frail	72 (44.4)	0.30	0.80 (0.53, 1.21)	0.90	0.94 (0.59, 1.50)	0.93
Not frail	78 (48.2)		(Reference)		(Reference)	
Cognitive impairment	45 (27.8)	0.84	0.96 (0.62, 1.49)	0.84	1.39 (0.84, 2.31)	0.20
Social isolation	22 (13.6)	0.11	1.67 (0.89, 3.15)	0.11	2.13 (1.05, 4.29)	<0.05
Impaired vision	52 (32.1)	0.75	0.93 (0.61, 1.43)	0.75	1.20 (0.75, 1.93)	0.45
Impaired hearing	51 (31.5)	0.22	1.32 (0.85, 2.05)	0.21	1.44 (0.89, 2.34)	0.14
Depression	37 (22.8)	0.11	0.69 (0.44, 1.10)	0.12	0.71 (0.40, 1.26)	0.24
**>=75 (*****N*** **= 412)**						
Frailty						
Frail	17 (14.7)	<0.01	0.63 (0.32, 1.25)	0.99	0.56 (0.23, 1.41)	0.97
Pre-frail	54 (46.6)		0.39 (0.24, 0.63)	<0.01	0.33 (0.18, 0.59)	<0.01
Not frail	45 (38.8)		(Reference)		(Reference)	
Cognitive Impairment	44 (37.9)	<0.01	0.55 (0.35, 0.85)	<0.01	0.67 (0.40, 1.11)	0.12
Social isolation	6 (5.2)	<0.05	0.44 (0.18, 1.07)	0.07	0.38 (0.14, 0.99)	<0.05
Impaired vision	30 (25.9)	0.32	0.79 (0.49, 1.27)	0.33	0.85 (0.49, 1.48)	0.56
Impaired hearing	52 (44.8)	0.34	0.81 (0.53, 1.25)	0.35	0.84 (0.52, 1.35)	0.47
Depression	28 (24.1)	0.69	0.91 (0.55, 1.49)	0.69	0.94 (0.51, 1.75)	0.85

*M1, Unadjusted logistic regression model for each geriatric element individually (main independent variables): frailty, cognitive impairment, social isolation, sensory impairments (visual and hearing), depression; M2, M1 additionally adjusted for risk scores (CHA_2_DS_2_VASC, HAS-BLED), education, insurance, atrial fibrillation type, AFEQT score, hyperlipidemia, alcohol abuse, anemia, chronic kidney disease, aspirin use, fall in past 6 months, IADL, provider type, frailty, and cognitive impairment*.

## Discussion

Over 80% of SAGE-AF participants were prescribed an OAC, a rate higher than has been reported in older cohorts but consistent with more recent data (33). This higher rate may also be explained by differences in the eligibility criteria used by our study, which required that participants not have active bleeding or other contraindications to OAC.

Although an increasing number of patients with AF are prescribed DOACs, VKAs are frequently used for stroke prevention (34). VKAs place a high burden on older patients, with dietary and lifestyle restrictions, frequent lab monitoring, and frequent dosage adjustments. We observed that 44% of anticoagulated study participants were treated with DOACs, a rate lower than has been reported in Europe and some specialized US centers (33), but slightly higher than what was reported by the IMS Health National Disease and Therapeutic Index (38%) (35). Consistent with national prescription rates, we observed that apixaban and rivaroxaban were the most commonly prescribed DOACs. Study region and prescriber type (cardiac electrophysiologist vs. other) were associated with type of OAC used for AF (Table 1).

Clinical trials and meta-analyses have suggested that DOACs may be safer than VKA in older trial participants (36, 37); however, many older patients were excluded from these studies based on commonly occurring comorbidities. The decision of VKA vs. DOACs in “real-world” patients remains a conundrum, since advanced age, and common comorbidities (e.g., renal impairment) increase the risk of adverse events from both agents (38). Moreover, in contrast to VKA, reversal agents for DOACs were not widely available during the study period, an important consideration for many providers since older patients with AF are at high risk for falls and traumatic bleeding (39).

Prior studies have shown that geriatric conditions, including cognitive impairment, frailty, and depression, are associated with a higher odds of not being prescribed OAC despite being eligible (40, 41); however, few studies have examined relations between geriatric conditions and type of OAC selected (42, 43). We hypothesized that older, frailer participants affected by a greater burden of geriatric conditions would be prescribed VKA, since these conditions are associated with renal impairment, polypharmacy, and other age-related factors that affect DOAC treatment.

Our finding that pre-frailty status (1–2 impairments) was associated with lower odds of being treated with a DOAC supports this hypothesis. Although the association between frailty status (more than 2 impairments) and greater odds of DOAC did not achieve statistical significance, the direction of the association was the same as pre-frailty (Table 2). We suspect the lack of statistical significance may have related to the smaller number of participants in the frail (vs. pre-frail) category. Furthermore, we observed a similar but statistically more robust finding among participants aged 75 years and older, but not among younger participants (65–74 years old). This suggests that the impact of geriatric conditions on OAC prescribing may be greatest among individuals older than 75 years of age. Furthermore, social isolation was associated with a 2-fold higher rate of DOAC use among younger patients. This relationship may be explained by the benefits of social support for handling the burden of monitoring and clinical follow-up required to manage VKA. However, among the oldest participants in our cohort, social isolation was associated with 62% lower odds of DOAC receipt. This counter-intuitive finding may be explained by AF patients or their providers perceiving frequent OAC clinic nurse evaluations and/or laboratory monitoring as a potential benefit for socially isolated elders.

Our findings have important potential clinical implications. Since AF patients in the pre-frail category were more likely to receive VKA, previously published secondary data analyses suggesting that DOACs have a favorable safety profile may suffer from unmeasured confounding (43). Second, patients receiving OAC had high rates of geriatric conditions, placing these individuals at elevated risk for OAC complications. In contrast to patients on VKA, patients receiving DOACs for AF are infrequently followed by anticoagulation clinics, nor do they systematically receive OAC education. Anticoagulation clinics support providers by helping to manage AF patients and perform routine drug safety and laboratory monitoring; however, these critical care pathways are not routinely made available to DOAC-treated patients.

Our findings also suggest that social isolation may influence OAC prescribing patterns and that the role of social isolation may differ among younger and older patients. Future studies should explore whether or how physicians and patients include the patient's social circumstances into conversations about treatment decision-making. Efforts to integrate support of DOAC-treated patients into traditional OAC clinics appear well-founded. Studies are needed to demonstrate that such supportive care improves patient outcomes.

The strengths of our study include the geographic diversity of the study cohort, inclusion of older AF patients with a high degree of comorbidity, and high rates of OAC use. Furthermore, the comprehensive assessment of factors associated with aging used validated and publicly available instruments that can be utilized in an office visit. Study limitations include the cross-sectional nature of our analysis. Information was not available about each participant's history or duration of exposure to OAC, and participants may have recently switched from DOAC to VKA or VKA to DOAC prior to study enrollment. However, prior studies show low overall rates of switching after initial OAC prescription, particularly among DOAC-treated patients (42, 44). Importantly, we had limited power to evaluate the associations between geriatric elements and use of DOACs among participants in Georgia, and thus cannot conclude whether the observed point estimates for these associations are statistically significant. Further research in other large, diverse samples are needed to validate our findings, especially considering the regional variation in OAC prescribing observed in our study.

## Conclusions

In a well-characterized, diverse sample of older patients with AF treated with OAC, we observed that the rates of readily assessed geriatric conditions were high and that several geriatric conditions, including pre-frailty and social isolation, were associated with a lower likelihood of receiving a DOAC. In light of guideline changes favoring DOACs over VKA for safety and effectiveness (5), additional efforts to provide tailored OAC education and support to frail and socially isolated patients may be necessary.

## Data Availability Statement

The datasets generated for this study will not be made publicly available as datasets contain sensitive, confidential information about study participants.

## Ethics Statement

The studies involving human participants were reviewed and approved by University of Massachusetts Medical School Institutional Review Board, Boston University Institutional Review Board, and Mercer University Institutional Review Board. The patients/participants provided their written informed consent to participate in this study.

## Author Contributions

DM had full access to all data in the study and takes responsibility for the integrity of the data and the accuracy of the data analysis. DM, CK, MW, DP, HA, RG, JG, and JS contributed to the conception and design of the study. DL, MW, FM, and JS organized the database. DL and BB performed the statistical analysis. All authors had access to the data and had a role in writing the manuscript.

### Conflict of Interest

DM has received research support from Apple Computer, Bristol-Myers Squibb, Boehringher-Ingelheim, Flexcon, Pfizer, Samsung, Philips Healthcare, Biotronik, and has received consultancy fees from Bristol-Myers Squibb, Pfizer, Flexcon, Boston Biomedical Associates, and Samsung. The remaining authors declare that the research was conducted in the absence of any commercial or financial relationships that could be construed as a potential conflict of interest.
