# Supplementary material for: Geriatric Conditions and Prescription of Vitamin K Antagonists vs. Direct Oral Anticoagulants Among Older Patients With Atrial Fibrillation: SAGE-AF
Source: Front Cardiovasc Med. 2019 Oct 30;6:155. doi: 10.3389/fcvm.2019.00155 (PMC6831524; doi:10.3389/fcvm.2019.00155)
Supplement: Supplementary file 1 [file Table_1.DOCX]

**Supplemental Data**

**Supplemental Table 1.** **Characteristics of older adults with atrial fibrillation on oral anticoagulation according to type of oral anticoagulation further stratified by geographic area of enrollment: SAGE-AF, 2016-2018**

|  | **Massachusetts**  **N=818** | | | **Georgia**  **N=246** | | |
| --- | --- | --- | --- | --- | --- | --- |
| **Characteristic** | **DOAC**  **N=278**  **n (%)** | **VKA**  **N=540**  **n (%)** | **p-value** | **DOAC**  **N=188**  **n (%)** | **VKA**  **N=58**  **n (%)** | **p-value** |
| ***Demographic Characteristics*** | | | | | | |
| **Age**  65-74 years  75-84 years  85 years or older | 162(58.3)  94 (33.8)  22 (7.9) | 244 (45.2)  196 (36.3)  100 (18.5) | <0.001 | 89 (47.3)  84 (44.7)  15 (8.0) | 24 (41.4)  27 (46.6)  7 (12.1) | 0.56 |
| **Female** | 129(46.4) | 270 (50.0) | 0.33 | 99 (52.7) | 27 (46.6) | 0.42 |
| **Race/Ethnicity**  White | 247(88.9) | 481 (89.1) | 0.92 | 133(70.7) | 41 (70.7) | 0.99 |
| **Marital Status ^a^**  Married or living as married  Not Married | 166(59.7)  110(39.6) | 299 (55.4)  234 (43.3) | 0.40 | 100 (53.2)  83 (44.2) | 28 (48.3)  27 (46.6) | 0.59 |
| **Education ^b^**  High school/GED or less  Some College  College graduate  Graduate Degree | 14 (5.0)  102(36.7)  53 (19.1)  107(38.5) | 35 (6.5)  273 (50.6)  78 (14.4)  146 (27.0) | <0.01 | 31 (16.5)  107 (56.9)  22 (11.7)  23 (12.2) | 10 (17.2)  37 (63.8)  5 (8.6)  4 (6.9) | 0.71 |
| **Insurance status ^c^**  Commercial/HMO/PPO  Medicare  Other | 51 (18.4)  194(69.8)  33 (11.9) | 125 (23.2)  371 (68.7)  43 (8.0) | 0.12 | 5 (2.7)  156 (83.0)  26 (13.8) | 3 (5.2)  51 (87.9)  3 (5.2) | 0.18 |
| ***Clinical Characteristics*** | | | | | | |
| **AF Type**  Paroxysmal  Persistent  Permanent  Unknown | 163(58.6)  71 (25.5)  17 (6.1)  27 (9.7) | 265 (49.1)  161 (29.8)  50 (9.3)  64 (11.9) | 0.06 | 135 (71.8)  47 (25.0)  2 (1.1)  4 (2.1) | 37 (63.8)  18 (31.0)  0 (0.0)  3 (5.2) | 0.34 |
| **CHA_2_DS_2_VASC score (M, SD)** | 4.1 (1.6) | 4.6 (1.6) | <0.001 | 4.7 (1.6) | 4.9 (1.5) | 0.27 |
| **HAS-BLED score (M, SD)** | 2.9 (1.0) | 3.0 (1.0) | 0.06 | 2.7 (1.0) | 2.6 (0.9) | 0.47 |
| **AFEQT score (M, SD)** | 79.0(18.3) | 81.3(16.6) | 0.08 | 77.1 (19.9) | 72.9(17.9) | 0.13 |
| **Bothered ^d^ by ≥1 AF symptom in the past 4 weeks** | 30 (10.8) | 50 (9.3) | 0.49 | 31 (16.7) | 5 (8.6) | 0.11 |
| **Medical History**  Acute Myocardial Infarction  Alcohol abuse/dependency  Anemia  Bleeding  Chronic kidney disease  Chronic lung disease  Diabetes  Heart failure  Hyperlipidemia  Hypertension  Implantable cardiac device  Peripheral vascular disease  Stroke | 46 (16.6)  105 (37.8)  59 (21.2)  60 (21.6)  57 (20.1)  66 (23.7)  72 (25.9)  77 (27.7)  211 (75.9)  240 (86.3)  73 (26.3)  38 (13.7)  20 (7.2) | 108 (20.0)  155 (28.7)  191 (35.4)  101 (18.7)  177 (32.8)  130 (24.1)  146 (27.0)  217 (40.2)  444 (82.2)  495 (91.7)  159 (29.4)  88 (16.3)  63 (11.7) | 0.23  <0.01  <0.001  0.33  <0.01  0.92  0.73  <0.01  <0.05  <0.05  0.34  0.32  <0.05 | 43 (22.9)  60 (31.9)  63 (33.5)  37 (19.7)  58 (30.9)  57 (30.3)  68 (36.2)  82 (43.6)  143 (76.1)  175 (93.1)  90 (47.9)  19 (10.1)  20 (10.6) | 15 (25.9)  13 (22.4)  21 (36.2)  11 (19.0)  18 (31.0)  15 (25.9)  20 (34.5)  33 (56.9)  51 (87.9)  56 (96.6)  44 (75.9)  5 (8.6)  4 (8.6) | 0.64  0.16  0.71  0.90  0.98  0.51  0.81  0.07  <0.05  0.31  <0.01  0.74  0.65 |
| **Creatinine (mg/dL) M (SD)** | 1.0 (1.0) | 1.2 (1.1) | <0.01 | 1.2 (0.5) | 1.1 (0.4) | 0.21 |
| **Hemoglobin (g/dL) M (SD)** | 13.5 (1.7) | 13.1 (1.9) | <0.05 | 12.3 (2.0) | 12.6 (1.4) | 0.37 |
| ***Treatment Characteristics*** | | | | | | |
| **Aspirin** | 69 (24.8) | 177 (32.8) | <0.05 | 35 (18.6) | 27 (46.6) | <0.001 |
| **Clopidogrel** | 10 (3.6) | 15 (2.8) | 0.52 | 20 (10.6) | 10 (17.2) | 0.26 |
| **Overall Treatment Satisfaction**  Extremely Satisfied  Very Satisfied  Somewhat satisfied  Satisfied or less than satisfied | 125(45.3)  86 (31.2)  37 (13.4)  28 (10.1) | 264(49.1)  170(31.6)  53 (9.9)  51 (9.5) | 0.45 | 78 (41.5)  61 (32.5)  24 (12.8)  25 (13.3) | 22 (37.9)  17 (29.3)  11 (19.0)  8 (13.8) | 0.70 |
| ***Psychosocial Characteristics*** | | | | | | |
| **Fall in past 6 months** | 36 (13.0) | 122 (22.6) | <0.01 | 55 (29.3) | 21 (36.2) | 0.32 |
| **Anxiety ^e^** | 61 (21.9) | 120(22.2) | 0.93 | 54 (28.7) | 17 (29.3) | 0.93 |
| **Living Alone** | 74 (26.6) | 153 (28.3) | 0.60 | 54 (28.7) | 14 (24.1) | 0.49 |
| **Independence (IADLs)** | 6.9 (0.6) | 6.7 (1.0) | <0.01 | 6.7 (1.1) | 6.3 (1.6) | 0.17 |
| **Confident in physician interactions ^f^** | 181(66.1) | 336(63.6) | 0.50 | 121 (68.0) | 38 (70.4) | 0.74 |
| **Practice Type**  Cardiologist  EP  Internist | 139(50.0)  131(47.1)  8 (2.9) | 349(64.6)  175(32.4)  16 (3.0) | <0.01 | 0 (0.0)  188(100.0)  0 (0.0) | 0 (0.0)  58(100.0)  0 (0.0) | NA |

**Legend.** a: n=17 missing Marital Status; b: n=17 missing Education; c: n=3 missing Insurance Status; d: quite/extremely/very bothered with symptoms; e: anxiety GAD7<=5;.f: defined based on a PEPPI score of greater than 45
